# Assessment of SARS-CoV-2 Anti-Spike IgG Antibody in Women and Children in Madinah, Saudi Arabia: A Single-Center Study

**DOI:** 10.3390/ijerph18199971

**Published:** 2021-09-22

**Authors:** Waleed H. Mahallawi, Nadir A. Ibrahim, Ammar S. Aljohani, Ehab A. Shaikh, Rayan H. Nafe, Anas M. Khan, Walaa A. Mumena

**Affiliations:** 1 Medical Laboratory Technology Department, College of Applied Medical Sciences, Taibah University, P.O. Box 344, Madinah 42353, Saudi Arabia; naibrahim@taibahu.edu.sa (N.A.I.); ammar.aljohani30@gmail.com (A.S.A.); Anas-malik@hotmail.com (E.A.S.); rayan.h.n34@gmail.com (R.H.N.); 2 KING Salman Medical City, General Directorate of Health Affairs of Madinah, Ministry of Health, Madinah 42353, Saudi Arabia; anaskhan4060@gmail.com; 3 Department of Clinical Nutrition, College of Applied Medical Sciences, Taibah University, P.O. Box 344, Madinah 42353, Saudi Arabia; wmumena@taibahu.edu.sa

**Keywords:** children, women, seropositivity, non-reported COVID-19 infection, SARS-CoV-2, predictor

## Abstract

Background: Coronavirus disease 2019 (COVID-19), caused by severe acute respiratory syndrome coronavirus 2 (SARS-CoV-2). Information on the prevalence of SARS-CoV-2 antibodies in women and children in Madinah has been limited. The current study aimed to evaluate SARS-CoV-2 IgG seropositivity among women and children at Madinah Maternity and Children’s Hospital. Methods: In this cross-sectional study, 579 participants were recruited between January and April 2021 from Madinah Maternity and Children’s Hospital, Saudi Arabia. Data concerning age, sex (for children), blood group, and height and weight (for women) were collected from the hospital database. SARS-CoV-2 anti-spike (anti-S) IgG antibodies were detected by enzyme-linked immunosorbent assay (ELISA). Results: Over 58% of children (*n* = 195), including 60% of children ≤ 1 year (*n* = 75), and 50.2% (*n* = 124) of women were SARS-CoV-2 anti-S IgG seropositive. Significantly higher anti-S IgG levels were observed in children than in women (0.78 ± 1.05 vs. 0.65 ± 0.98, *p* = 0.008). Compared with women, children had higher odds of high SARS-CoV-2 anti-S IgG levels (odds ratio: 1.41; 95% confidence interval: 1.01–1.97; *p* = 0.041). No significant associations were observed for anti-S IgG levels with age in women or children or with body mass index among women. Conclusion: Non-reported COVID-19 infections were more prevalent among children than women, and non-reported COVID-19 infections children represent a viral transmission risk; therefore, increased screening, especially among school-aged children, may represent an important COVID-19 preventive control measure.

## 1. Introduction

In December 2019, people in China were diagnosed with an unknown respiratory disease, later identified as coronavirus disease 2019 (COVID-19), which is caused by the novel severe acute respiratory syndrome coronavirus 2 (SARS-CoV-2) [1]. The outbreak spread to Europe and the Americas in mid-March 2020, developing into a worldwide health emergency (https://www.who.int/emergencies/diseases/novel-coronavirus-2019/events-as-they-happen, accessed on 18 July 2021).

The first case of COVID-19 infection in the Kingdom of Saudi Arabia was detected in the Eastern Province in March 2020. The affected individual was a Saudi Arabian citizen who had traveled to an affected region in Iran. Thereafter, sporadic cases were identified in other regions [2].

Real-time polymerase chain reaction (RT-PCR) has been acknowledged as the gold standard for confirming the presence of SARS-CoV-2 infection; however, viral detection is restricted by the transient nature of RNA, and concerns have been raised regarding the unsatisfactory sensitivity of this method [3], which may result in the misdiagnosis of SARS-CoV-2 infections, especially among subclinical or non-reported COVID-19 infections cases. Population-based serosurveys measuring anti-SARS-CoV-2 antibodies offer one method for approximating infection rates and monitoring the progress of the epidemic [4]. By identifying individuals who have established anti-viral antibodies, immunoassay, such as enzyme-linked immunosorbent assay (ELISA), can provide a more thorough understanding of the SARS-CoV-2 prevalence [5]. In fact, the current study is important to observe and trace COVID-19 incidence and recognize the frequency of or non-reported COVID-19 infections individuals in the community. In addition, to measure the prevalence of SARS-CoV-2, which will help in trace and contain disease and the viral spread especially in the highly affected regions.

Children with COVID-19 rarely present with severe respiratory symptoms and frequently remain asymptomatic [6], whereas adults more commonly develop respiratory symptoms of variable severity [7]. Children younger than 10 years with no identified SARS-CoV-2 exposure have been diagnosed with asymptomatic SARS-CoV-2 infection [8]. In contrast with adults, children with COVID-19 typically experience a mild illness with a good prognosis [9]. Interestingly, a recent study found that there are cross-reactive epitopes mediated T cell response induced by hCoVs and SARS-CoV-2 infection, making children more likely to have asymptomatic or mild clinical symptoms when compared to adults because seasonal hCoVs infections are more frequent in children than adults [10].

A seroprevalence study conducted among blood donors revealed that blood group O donors have a lower seroprevalence than those in blood Group A [11], and the same pattern was observed among blood donors in Saudi Arabia [12]. Another seroprevalence study showed that COVID-19 was more commonly identified as a non-reported COVID-19 infection among pregnant women, and the use of immunoassays can detect non-laboratory confirmed infections [13]. One study reported a nearly 50% anti-SARS-CoV-2 antibody seroprevalence among children who were asymptomatic COVID-19 infection, and the transmission rates to children from a laboratory-confirmed SARS-CoV-2–infected family member were 35% [14].

Knowing the seroprevalence of SARS-CoV-2 infections among asymptomatic COVID-19 infection women and children is important for developing an informed pandemic response. In the current study, we aimed to screen women and children with no known history of SARS-CoV-2 infection during regular follow-up appointments at Madinah Maternal and Children’s Hospital using an ELISA to identify the seroprevalence status. In fact, the current study is important to observe and trace COVID-19 incidence and recognize the frequency of non-reported COVID-19 infection individuals in the community. In addition, to measure the prevalence of SARS-CoV-2, which will help in trace and contain disease and the viral spread especially in the highly affected regions (the seroprevalence of SARS-CoV-2 IgG antibodies among asymptomatic blood donors in Saudi Arabia). 

## 2. Materials and Methods

### 2.1. Sample Collection

Data from 579 participants were collected between January and April 2021. The minimum numbers of participants necessary for this study were 162 women and 162 children, based on the equation suggested by Charan and Biswas [15], using alpha = 0.05, an expected proportion of 30% positive SARS-CoV-2 anti-spike (anti-S) IgG cases, and a precision of 5%. 

Participants were recruited through the outpatient clinics in Madinah Maternity and Children’s Hospital, Saudi Arabia (247 women and 332 children) who were on follow-up during the time of conducting the study. The hospital is the biggest in Madinah region and accounted as a specialized hospital that receive patients from Madinah city and the surroundings areas. Additionally, the hospital accepts patients’ referrals from the free governmental primary health centers and private hospitals. In terms of children ages, the hospital receives children patients from birth up to 17 years. Nevertheless, no age limit for the women patients. Generally, the majority of the women patients were in childbearing age and some were on follow-up in gynecological clinics. We only included those with no previous history of SARS-CoV-2 infection. Upon personal survey and comparison against the Ministry of Health database, we excluded any individuals with a positive infection history. Additionally, those who received any COVID-19 vaccines were also excluded. 

All adult patients signed informed consent, and the parents of children patients signed informed consent on behalf of their children before conducting the study. The study was approved by the ethical committee of the College of Applied Medical Sciences (Certificate No. 2021/96/117/MLT).

### 2.2. Assessment of Outcome: SARS-CoV-2 Anti-Spike IgG Antibody Detection

After informed consent was obtained from each participant, blood samples were collected in 4-mL EDTA vacutainer plasma tubes and immediately transported to the Virology Research Laboratory for further processing. Samples were centrifuged for 10 min at 2000 RPM within one hour of collection using a routine centrifuge. Plasma was then transferred to plastic screw-cap vials and frozen at −20 °C until analysis.

### 2.3. Enzyme-Linked Immunosorbent Assay (ELISA)

Indirect ELISA for the detection of SARS-CoV-2 anti-S IgG antibodies was performed by using the commercial SARS-CoV-2 IgG ELISA Kit (BGI Europe A/S) (the kit fits to using serum and plasma), according to the manufacturer’s instructions. The test specificity for IgG antibody detection is 98.38%, and the sensitivity for IgG antibody detection is greater than 98.71%, according to the manufacturer (https://www.bgi.com/global/molecular-genetics/covid-19-antibody-detection-kit-elisa/ accessed on 29 July 2021). In brief, 96-well (pre-coated with purified SARS-CoV-2 viral antigen) ELISA plates were used. Two wells of the plate were reserved for the negative control (for cutoff calculations), one well contained the positive control, and one well was left blank; 100 µL of positive control and negative control were added to the designated wells without dilution, and no liquid was added to the blank well. For the remaining wells, 10 µL of the plasma was added to each well along with 100 µL of sample diluent buffer, and the plate was incubated at 37 °C for 30 min. The plates were then washed five times using a semi-automated ELISA washer, and 100 µL of horseradish peroxidase-labeled anti-human IgG antibody was added to each well and incubated for 20 min at 37 °C. The plates were then washed five times, and 50 µL each of Substrates A and B were added to each well and incubated in the dark for 10 min at 37 °C. After the color development was completed, 50 µL stop solution was added to each well. Finally, the optical densities were determined at 450 nm. The cutoff values for anti- SARS-CoV-2 IgG antibody detection were calculated (0.1 + mean absorbance of the two negative controls).

### 2.4. Assessment of Predictors: Age, Sex, Blood Group, and Weight Status

Data concerning age, sex of children, and blood group of all participants were collected from the system. Data for the heights and weights of women ≥20 years were obtained from patients’ record. These data were used to calculate the body mass index (BMI) values for participating women to assess their weight status, which was categorized according to the recommended World Health Organization (WHO) criteria, as follows: underweight, BMI < 18.5 kg/m^2^; healthy weight, BMI between 18.5 kg/m^2^ and <24.9 kg/m^2^; overweight, BMI between 25.0 kg/m^2^ and 29.9 kg/m^2^; and obese, BMI ≥ 30.0 kg/m^2^.

### 2.5. Statistical Analysis

Data were analyzed using the Statistical Package for Social Sciences (SPSS 20, SPSS Inc., Chicago, IL, USA). Categorical variables are described as the frequency and percent (%), whereas continuous variables are described as the mean ± standard deviation (SD) or the median (interquartile range [IQR]). Fisher’s exact test was used to assess associations between two categorical variables. The Shapiro–Wilk test was used to assess the normality of distribution for continuous variables (age and SARS-CoV-2 anti-S IgG antibody). The Mann–Whitney U test was used to compare mean levels of SARS-CoV-2 anti-S IgG antibodies among women and children to compare the mean ages of women and children between those with negative and positive SARS-CoV-2 serological status. Spearman’s correlation coefficient was calculated between age and SARS-CoV-2 anti-S IgG antibody. A logistic regression analysis was performed to estimate the odds ratios (ORs) for SARS-CoV-2 serological status among the different groups. For the regression analysis, the categorical variables were coded as follows: SARS-CoV-2 serological status (negative = 0, positive = 1); sex (male = 1, female = 2); nationality (Saudi = 1, non-Saudi = 2); weight status (underweight = 1, healthy weight = 2, overweight = 3, obese = 4); blood group (A = 1, B = 2, AB = 3, O = 4). All tests were two-tailed with a significance level of 95%.

## 3. Results

### 3.1. General Characteristics

A total of 579 participants were included in the final analysis of this study (247 women and 332 children) after excluding 19 participants (3.18%) due to missing data. The mean age among women was 33.4 ± 9.8 years, with 86.2% (*n* = 213) Saudi nationals. The blood group distribution for women was 48.2% (*n* = 119) Type O, 31.6% (*n* = 78) Type A, 17.4% (*n* = 43) Type B, and 2.8% (*n* = 7) Type AB. Only 26.7% (*n* = 65) of the women included in this study were within the healthy weight range. The mean age among children was 5.42 ± 4.23 years, including 22.6% (*n* = 75) ≤ 1 year and 22.7% (*n* = 17) newborn. Over half of the children included in the study were males (53.6%, *n* = 178), and 46.4% (*n* = 154) were females. The blood group distribution for children was 45.0% (*n* = 152) Type O, 30.7% (*n* = 102) Type A, 19.0% (*n* = 63) Type B, and 4.5% (*n* = 15) were Type AB. A detailed description of the characteristics of the study participants are provided in Table 1.

### 3.2. Prevalence of Anti-S IgG Antibody to SARS-CoV-2 among Participants

We evaluated the presence of the SARS-CoV-2 anti-S IgG antibody using ELISA, which showed that 50.2% (*n* = 124) of women and 58.7% (*n* = 195) of children were seropositive for SARS-CoV-2 antibodies. Prevalence among newborns specifically (ages since born until 27 days) was 4.10% (*n* = 8). The median level of anti-S IgG antibody among children was 0.15 (0.09–1.17 in OD unit), whereas the median level of anti-S IgG antibody among women was 0.11 (0.09–0.78 in OD unit). The mean level of anti-S IgG antibody among children was significantly higher than that for women (0.78 ± 1.05 vs. 0.65 ± 0.98, *p* = 0.008). Compared with women, children had higher odds of having higher SARS-CoV-2 anti-S IgG antibody to (OR: 1.41; 95% confidence interval: 1.01 to 1.97; *p* = 0.041). Among the 22.6% of children ≤ 1 year (*n* = 75), 60.0% were SARS-CoV-2 anti-S IgG antibody seropositive.

### 3.3. Association between SARS-CoV-2 Serological Status and the Characteristics of Participants

We performed univariate analysis to identify associations between SARS-CoV-2 serological status and the characteristics of participants, as presented in Table 2. The mean age of women with negative SARS-CoV-2 serological status was significantly lower than the mean age of women with positive SARS-CoV-2 serological status (31.5 ± 8.55 years vs. 35.4 ± 10.6 years, *p* = 0.004). All other characteristics of the women included in this study were similar. The characteristics of the children in this study did not differ between groups according to SARS-CoV-2 serological status (negative vs. positive).

### 3.4. Correlation between SARS-CoV-2 Anti-S IgG Antibody and Age

**Figure 1:**
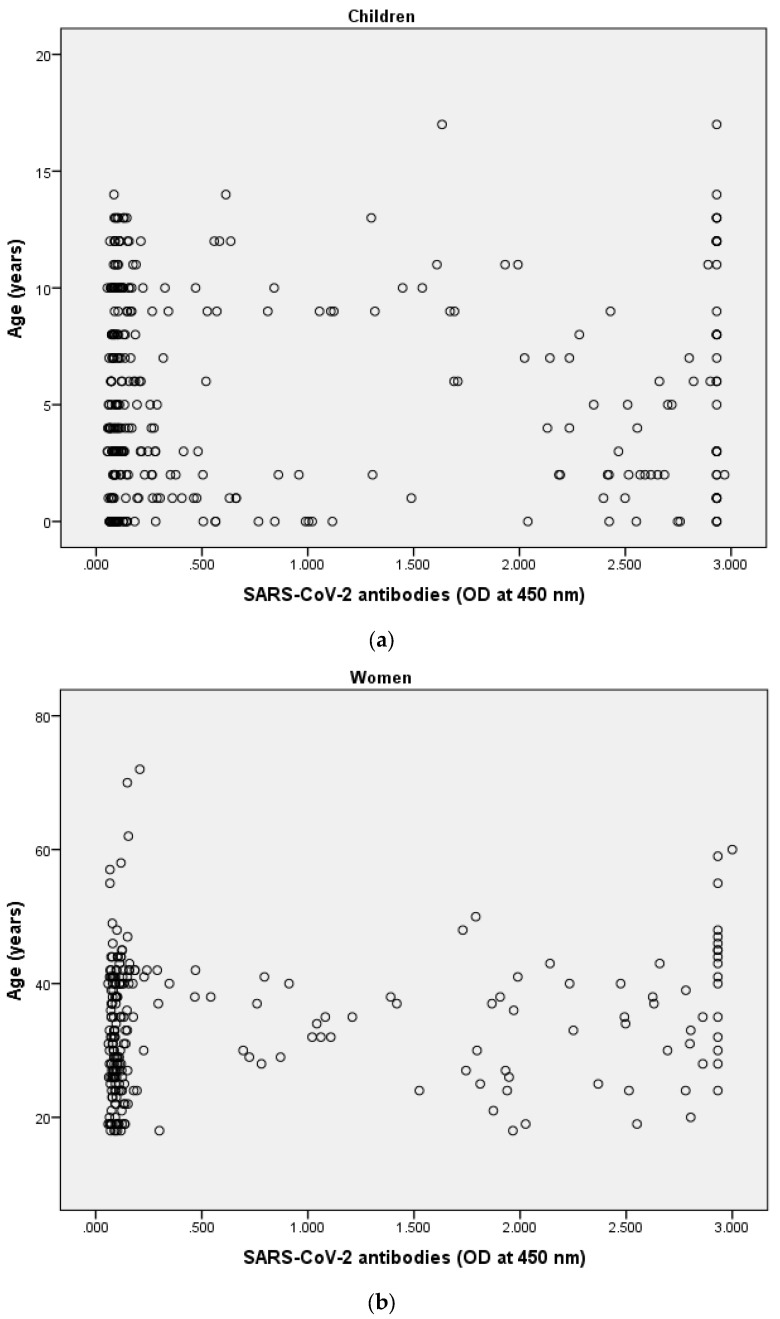
The result showed no correlation between antibody levels and age in children (**a**), r_s_ = 0.01, *p* = 0.857). By contrast, a weak positive correlation was observed between anti-S IgG antibody levels and age in women (r_s_ = 0.19, *p* = 0.004, (**b**)).

Spearman’s correlation analyses were performed between SARS-CoV-2 anti-S IgG antibody and age in both women and children. The result showed no correlation between antibody levels and age in children (Figure 1a, r_s_ = 0.01, *p* = 0.857). By contrast, a weak positive correlation was observed between anti-S IgG antibody levels and age in women (r_s_ = 0.19, *p* = 0.004, Figure 1b).

Data obtained from the logistic regression analysis showed that higher women’s age increased the odds of SARS-CoV-2 anti-S IgG antibody status (odd ratio (OR) = 1.04; 95% confidence Interval = 1.02 to 1.07; *p* = 0.002). None of the other variables among women and children predicted SARS-CoV-2 anti-S IgG antibody status (Table 3).

## 4. Discussion

A large proportion of the children examined in this study were positive for the SARS-CoV-2 anti-S IgG, and the mean level of anti-S IgG antibody among children was significantly higher than that among women. Compared with women, children had higher odds of having higher SARS-CoV-2 anti-S IgG antibody levels. Among the 22.6% of children ≤ 1 year, 60% were seropositive for the SARS-CoV-2 anti-S IgG antibody. No significant association was observed between anti-S IgG antibody levels and age or BMI among women. Similarly, no associations were found between anti-S IgG antibody levels and age in children.

Population-based serosurveys measuring anti-SARS-CoV-2 antibodies offer one method for approximating infection rates and monitoring the progress of the epidemic [4]. Using highly specific and sensitive immunoassays when perform serosurveys is critical to ensuring reliable and meaningful results [16]. Timely diagnosis and isolation, sufficient disease management, prevention strategies, and improved vaccine performance and delivery all contribute to controlling the spread of disease [6].

Saudi Arabia has reported more than half a million confirmed COVID-19 cases, with the most recent reports indicating 10,905 active cases, 481,225 recovered cases, and 8063 deaths. Our study was conducted in Madinah, Saudi Arabia, which has recorded 25,011 confirmed cases, 24,482 recovered cases, and 279 deaths. To date, 22,112,330 vaccine doses have been administered in Saudi Arabia (https://covid19.moh.gov.sa/, accessed on 17 July 2021).

A recent study has reported that approximately one-fifth of all blood donors in Madinah are seropositive for anti-SARS-CoV-2 IgG antibodies, all of whom were non-laboratory-confirmed COVID-19 donors [12].

In the current study, none of the participants had a history of confirmed SARS-CoV-2 infection; therefore, we assumed that seropositive individuals were likely non-reported COVID-19 infection patients. Asymptomatic individuals infected with the SARS-CoV-2 virus can perpetuate the continued transmission of the virus within the community and pose a significant threat to society, serving as a primary source of infection [17].

Until recently, the contributions of children and adolescents in the transmission of SARS-CoV-2 have been indeterminate. Evidence suggests that children are less vulnerable to infection and less likely to develop severe disease in response to infection [18].

We found that nearly 60% of children were seropositive for SARS-CoV-2 anti-S IgG antibody. This large antibody prevalence among children suggests a high proportion of COVID-19 infections. Therefore, children likely represent a silent source of SARS-CoV-2. Our results align with those of a study conducted in the USA, which showed that 68% of confirmed SARS-CoV-2–positive pediatric cases presented with no symptoms of fever, cough, or shortness of breath [19].

Several studies have been conducted examining the seroprevalence of SARS-CoV-2 antibodies. One study performed in South Korea showed a high seroprevalence rate among asymptomatic individuals (62%). Another study conducted on asymptomatic individuals in Chelsea, Massachusetts, revealed a moderate seropositivity rate (31.5%) [20,21].

Asymptomatic individuals, including children, are expected to spread COVID-19 throughout the community. Therefore, the application of infection control measures, such as social distancing and daily preventive habits, is advised for people of all ages to reduce infection spread, prevent the overburdening of health care facilities, and protect those with underlying medical conditions [22]. Our results revealed that the seropositivity rate in children is similar according to sex, with 61.8% seropositivity observed in males and 55.2% in females. This result is inconsistent with finding of previous study that reported higher seropositivity rate in boys than girls [23]. However, our previous study found a higher SARS-CoV-2 viral load (based on lower Ct values) among females compared with males [24]. The idea that sex might influence viral loads and the immunological response to infectious disease is not unexpected, and similar effects have been demonstrated for other viral infections [25].

We found that the mean level of anti-S IgG antibody among children was significantly higher than that among women (0.78 ± 1.05 vs. 0.65 ± 0.98, *p* = 0.008). Our finding is in agreement with a study that found neutralizing antibody against SARS-CoV-2 was inversely associated with age [26]. Additionally, our aligned with a new study, which defined greater neutralizing capability, and avidity of antibodies against the virus in children aged 1 to 10 years, in a cohort of subjects aged 1 to 24 years, early after recovery [27]. However, our results differ from those of a cohort study that showed higher anti-S IgG antibody levels in adults compared with children [18]. Moreover, we found an association between age and antibody levels among women. Therefore, considering higher antibody levels in women would be a possible predictor for them to experience a COVID-19 infection. However, previous studies have been conducted on laboratory-confirmed and hospitalized patients. Therefore, further investigations performed on asymptomatic individuals remain necessary.

We identified 22.6% of the children in our study as ≤1 year (*n* = 75), and 60% of this population were seropositive for SARS-CoV-2 anti-S IgG antibody. This high prevalence could be due to the transplacental transference of maternal antibodies to infants during pregnancy, although the possibility of infection cannot be excluded. However, that possible due to the cross-reactive exist between SARS-CoV-2 and HCoVs such as 229E, OC43, NL63, HKU1 [10]. Among the 22 mother/newborn dyads for which corresponding sera were obtainable, we found anti-S IgG antibodies in 91% of newborns from anti-S IgG–positive non-reported COVID-19 infection mothers (data not shown). Our results agree with those from a recent study that showed SARS-CoV-2 IgG antibodies could be detected in the cord blood obtained from 72 of 83 newborns (87%) delivered to mothers who were IgG antibody–positive at the time of delivery. Additionally, the study showed that maternal SARS-CoV-2 IgG antibodies could be transferred across the placenta after both asymptomatic and symptomatic infections during pregnancy [28].

SARS-CoV-2 infections of placental cells do not necessarily result in neonatal infections. One study showed that mothers positive for SARS-CoV-2 transferred the infection to a marginal number of neonates [29]. Consequently, no major neonatal respiratory diseases occurred. Several studies have reported on neonatal outcomes, and no severe adverse consequences have been witnessed among neonates born to laboratory-confirmed SARS-CoV-2–infected mothers [30,31,32].

Among the women who participated in this study, 88 were pregnant (35.63%), 35 were postpartum (14.17%), and 124 were non-pregnant (50.2%). The seropositivity rate did not differ significantly across these categories (data not shown).

Our results show variations in the seropositivity percentages associated with BMI among women. Healthy weight, overweight, and obese women presented with seropositivity rates of 52.3%, 40.7%, and 57.4%, respectively. Although the differences among these three categories were not significant, we identified higher seropositivity levels among obese women. Our findings are consistent with a previous study that reported obese individuals as having a 26% higher risk of contracting COVID-19 than normal-weight individuals [27]. Overweight and obesity are possible risk factors of COVID-19 and should be considered when planning COVID-19 pandemic monitoring strategies (Ji et al., 2021).

We also investigated the association between SARS-CoV-2 IgG antibody seroprevalence and blood groups and found no significant correlation among these variables. However, our recent study showed a higher seropositivity rate for the SARS-CoV-2 IgG antibody in asymptomatic male blood donors with Blood Type A [12].

To the best of our knowledge, our study is the first study in Saudi Arabia to investigate SARS-CoV-2 IgG seropositivity among women and children. However, this study might be limited by the sampling bias caused by the data recruited from a single hospital. In addition, official data concerning the viral strains and correlate that with the epidemic characteristic of the population were lacking. Although high temperature in Saudi Arabia could be a factor that influence the findings of this study, data from the United States found that lower temperatures was correlated with increased SARS-CoV-2 transmission [33]. Therefore, further investigation concerning this issue is recommended to be explored in future studies.

Non-reported COVID-19 infections were found to be more prevalent in children than in women, indicating that asymptomatic children represent a potential risk for increased risk of viral transmission and spread throughout the community. Consequently, increased screening measures among children, especially school-aged, may serve as an important preventive pandemic control measure. More serosurveys remain necessary to clarify the infection rate and verify vaccination coverage. Studies that compare data between men and women are also necessary to investigate potential sex differences among adults. Furthermore, investigations exploring the level of cellular immunity in asymptomatic individuals are necessary, as a recent study showed that patients with asymptomatic SARS-CoV-2 infection had a significantly increased natural killer (NK) cells fraction compared with the healthy, non-infected group, suggesting that NK cells may serve as an indicator of asymptomatic infections [34].

## Figures and Tables

**Table 1:** Characteristics of the study participants (*n* = 579).

Children (*n* = 332)	
Age in years, mean ± SD median (IQR)	5.42 ± 4.23 5.00 (2.00–9.00)
Children aged ≤ 1 year, *n* (%)	
Yes	75 (22.6)
No	257 (77.4)
Newborn, *n* (%)	
Yes	17 (22.7)
No	58 (77.3)
Sex, *n* (%)	
Male	178 (53.6)
Female	154 (46.4)
Nationality, *n* (%)	
Saudi	279 (84.0)
Non-Saudi	53 (16.0)
Blood group, *n* (%)	
A	102 (30.7)
B	63 (19.0)
AB	15 (4.50)
O	152 (45.8)
Women (*n* = 247)	
Age in years, mean *±* SD median (IQR)	33.4 ± 9.80 33.0 (26.0–40.0)
Nationality, *n* (%)	
Saudi	213 (86.2)
Non-Saudi	34 (13.8)
Blood group, *n* (%)	
A	78 (31.6)
B	43 (17.4)
AB	7 (2.80)
O	119 (48.2)
Weight status ^1^, *n* (%)	
Underweight	3 (1.36)
Healthy weight	57 (25.8)
Overweight	74 (33.5)
Obese	87 (39.4)

^1^*n* = 221.

**Table 2:** Associations of SARS-CoV-2 serological status and characteristics of participants (*n* = 579).

Children (*n* = 332)			
	Negative (*n* = 137)	Positive (*n* = 195)	*p*-Value
Age in years, mean ± SD Median (IQR)	5.36 ± 4.11 4.00 (2.00–9.50)	5.47 ± 4.32 5.00 (2.00–9.00)	0.963
Children aged ≤ 1 year, *n* (%)			
Yes	30 (40.0)	45 (60.0)	0.894
No	107 (41.6)	150 (58.4)	
Newborn, *n* (%)			
Yes	9 (52.9)	8 (47.1)	0.325
No	128 (40.6)	187 (59.4)	
Sex, *n* (%)			
Male	68 (38.2)	110 (61.8)	0.264
Female	69 (44.8)	85 (55.2)	
Nationality, *n* (%)			
Saudi	117 (42.1)	162 (58.1)	0.649
Non-Saudi	20 (37.7)	33 (62.3)	
Blood group, *n* (%)			
A	41 (40.2)	61 (59.8)	0.516
B	22 (34.9)	41 (65.1)	
AB	8 (53.3)	7 (46.7)	
O	66 (43.4)	86 (56.6)	
**Women (*n* = 247)**			
	**Negative** **(*n* = 123)**	**Positive** **(*n* = 124)**	***p*-Value**
Age in years, mean ± SD Median (IQR)	31.5 ± 8.55 30.0 (26.0–38.0)	35.4 ± 10.6 35.0 (27.0–42.0)	0.004 ^1^
Nationality, n (%)			
Saudi	107 (50.2)	106 (49.8)	0.356
Non-Saudi	14 (41.2)	20 (58.8)	
Blood group, *n* (%)			
A	32 (41.0)	46 (59.0)	0.434
B	22 (51.2)	21 (48.8)	
AB	5 (71.4)	2 (28.6)	
O	62 (52.1)	57 (47.9)	
Weight status ^2^, *n* (%)			
Underweight	2 (66.7)	1 (33.3)	0.076
Healthy weight	27 (47.4)	30 (52.6)	
Overweight	44 (59.5)	30 (40.5)	
Obese	35 (40.2)	52 (59.8)	

^1^ alpha = 0.05. ^2^ *n* = 221.

**Table 3:** Logistic regression analysis of SARS-CoV-2 anti-S IgG antibody status and participants’ characteristics (*n* = 579).

	OR	95% Confidence Interval	*p*-Value
**Children**			
Age, years	1.01	0.96 to 1.06	0.817
Sex			
Male	Refrence catogary		
Female	0.76	0.49 to 1.18	0.223
Nationality			
Saudi	Refrence catogary		
Non-Saudi			
Blood group			
A	Refrence catogary		
B	1.25	0.65 to 2.40	0.50
AB	0.59	0.20 to 1.75	0.339
O	0.88	0.53 to 1.46	0.610
**Women**			
Age, years	1.04	1.02 to 1.07	0.002 ^1^
Nationality			
Saudi	Refrence catogary		
Non-Saudi	1.48	0.71 to 3.09	0.292
Blood group			
A		Refrence catogary	
B	0.74	0.35 to 1.56	0.424
AB	0.31	0.06 to 1.69	0.176
O	0.71	0.40 to 1.26	0.243
Weight status			
Underweight		Refrence catogary	
Healthy weight	2.06	0.18 to 23.9	0.562
Overweight	1.38	0.12 to 15.8	0.80
Obese	2.70	0.24 to 30.8	0.42

^1^ alpha = 0.05.
